# Steroids in paediatric heart surgery: eminence or evidence-based practice?

**DOI:** 10.1007/s12055-018-0670-y

**Published:** 2018-04-03

**Authors:** Daniel Fudulu, Stafford Lightman, Massimo Caputo, Gianni Angelini

**Affiliations:** 1 Department of Cardiac Surgery, Bristol Heart Institute, Bristol, UK; 2 Henry Wellcome Laboratories for Integrative Neuroscience and Endocrinology, University of Bristol, Dorothy Hodgkin Building, Bristol, UK; 3 Department of Congenital Cardiac Surgery, Bristol Children’s Hospital, Bristol, UK

**Keywords:** Steroids, Clinical outcomes, Relative adrenal insufficiency

## Abstract

Steroids in paediatric heart surgery are given prophylactically to blunt the systemic inflammatory response induced by the extracorporeal circuit and to improve clinical outcomes. However, there is an ongoing controversy about the impact of steroids on clinical outcomes after paediatric heart surgery. The hypothalamic-pituitary-adrenal axis is the primary neuroendocrine system activated during the stress of surgery. Relative adrenal insufficiency can accompany paediatric heart surgery; therefore, perioperative steroid supplementation is still administered by some centres. The studies that investigate the hypothalamic-pituitary-adrenal axis physiology during surgery have many limitations, and it is unclear how to define what is adrenal insufficiency. In this review, we focus on discussing the available evidence for steroid use in paediatric cardiac surgery.

## Background

**Fig. 1:**
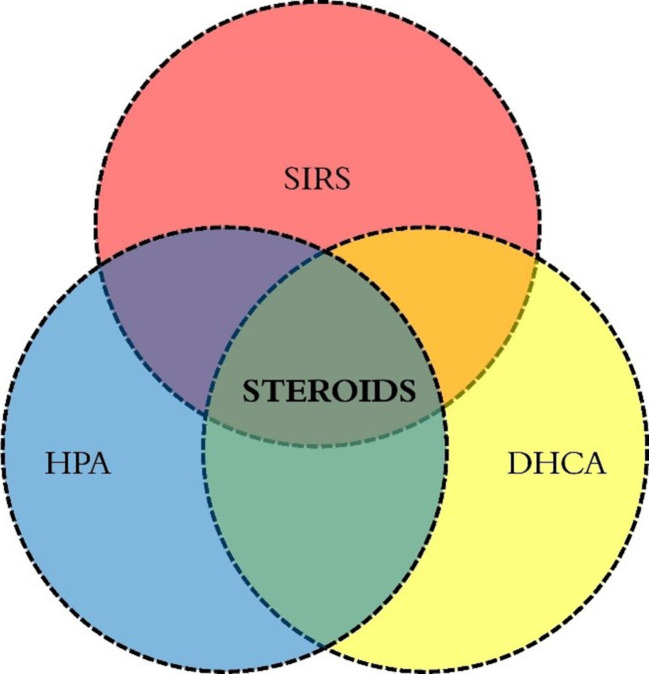
The main steroid indications in paediatric heart surgery

Most of the progress in cardiac surgery results from the discovery of heparin followed by the introduction of the cardiopulmonary by-pass machine that made possible open cardiac surgery [1]. However, quite early after its introduction in the 1950s, attempts were made to both understand and modulate the adverse effects of the extracorporeal circuit. Indeed, the first study of the effect of glucocorticoids in cardiac surgery was published by Replodge et al. in 1966. The authors looked at the effect of large doses of dexamethasone administered perioperatively in 20 children and adults undergoing heart surgery with the use of cardiopulmonary by-pass [2]. They found steroid recipients to have a lower beta-glucuronidase enzyme activity thus suggesting reduced lysosomal injury. However, there was no clear benefit in terms of clinical outcomes apart from noticing that the steroid recipient patients had a “better general appearance and state postoperatively”. Our current understanding of the response to surgery is that the contact of the blood with the extracorporeal circuit, the ischaemia-reperfusion injury, and endotoxemia induce a generalised immune system activation called the *systemic inflammatory response*. It is also believed that there is a balance between “good” inflammation that acts to prime the immune system and “bad” inflammation that could result in multiple end-organ failure and death [3]. In children, the surgical inflammatory response tends to be amplified due to their lower circulating blood volume compared to the size of the extracorporeal circuit and due to the frequent use of deep hypothermic arrest surgery and haemodilution [4]. According to several surveys of clinical practice, glucocorticoids are still widely used as a pharmacologic tool to modulate the inflammation [5]. Most of the centres do not administer steroids in every case but reserve their use for the most “high-risk” cases: neonates, deep hypothermic cardiac arrest surgery, and long cardiopulmonary by-pass [5]. Such indications vary widely between centres, and there is no consensus because of limited research in this area. Apart from this generalised, immune response to surgery, the stress of surgery is also accompanied by a neuroendocrine response. The primary neuroendocrine system activated during surgery is the hypothalamic-pituitary-adrenal axis (HPA) that produces the stress hormone *cortisol*. Therefore, another justification behind the use of steroids is to cover for the presumed perioperative relative adrenal insufficiency that can accompany cardiac surgery [6]. Finally, steroids are also administered to protect against brain injury during DHCA surgery. In the current review, we will discuss these three main areas of steroid use (Fig. 1). Another indication for steroid administration is immunosuppression after paediatric heart transplantation, but this area is not within the scope of this review.

## Methodology

We conducted a literature search using PubMed and MEDLINE databases. The search phrase included a combination of the following terms: “steroid”, “glucocorticoid”, “corticosteroid”, “dexamethasone”, “hydrocortisone”, “methylprednisolone”, “pediatric”, “paediatric”, “heart surgery”, “cardiac surgery”, “children”, “neonates”, “deep hypothermic circulatory arrest”, “adrenal”. The last search was conducted in November 2017. We identified additional articles by cross-referencing from author reference lists and published review papers. We have included all relevant articles assessing the effect of corticosteroids on inflammation, clinical outcomes, adrenal function, and brain injury during paediatric heart surgery with the use of cardiopulmonary by-pass.

## Less inflammation, better clinical outcomes?

Throughout the years, several studies measured the effect of glucocorticoids on markers of inflammation but most importantly tried to correlate the effect on clinical outcomes. Some studies found that steroids can blunt inflammation [7–14] while others found the opposite [15]. Most importantly, it is unclear to what degree this presumed “anti-inflammatory” effect also translates into better clinical outcomes [7, 10, 13]. Firstly, it is very difficult to characterise the *multifaceted* nature of the SIRS response whereby a variety of cytokines are activated and interact in a rather complex, unpredictable manner [16]. Secondly, we know that the use of steroids is subject to huge variability in the practice between centres and this is also reflected in the design of the studies that use various types of steroids, various regimens, or routes of administration thus making more difficult the correlation between inflammation and clinical outcomes [5, 17]. Thirdly, the available studies are small-sized and display a variability between the endpoints measured (e.g. inflammation or clinical outcomes measured as a primary or the secondary outcome). Finally, it is likely that a standard corticosteroid regimen given to all patients might not address the individual, host inflammatory response and it is likely that the approach to SIRS will have to be personalised rather one-for-all regimen [18].

There are several small size, randomised trials of steroids versus placebo conducted on children of various ages or just neonates [8–10, 19–21]. Their results are conflictive with some trials reporting benefit [8, 11, 19, 21] while others reporting no effect on clinical outcomes [7, 9, 13, 20]. The results of an ongoing trial on 190 neonates are still awaited and hopefully will give us more information [22]. Several studies focused at the effect of the route, dose, or timing of steroid administration. One small RCT study found no dose-effect of steroids on clinical outcomes [9]. Another RCT found no differences between the single intraoperative dose of steroid and double (pre-operative and intraoperative doses) [23]. One study investigated the effect of steroid administered intravenously versus steroid in prime and found no differences [24]. Due to the limitations of the randomised evidence described above, the resulting meta-analyses are of limited value too [25, 26]. Some of the studies cited above focused on neonate patients only. This is because this group is particularly vulnerable to CPB insult due to (1) a distinct inflammatory response, (2) because of an immaturity of the HPA axis that prevents them to mount an adequate stress response. A recent systematic review of the literature found no clear benefit of steroid use in this particular group of patients [27].

The use of steroids will continue to be a matter of intense debate until we have the results of large, multicentre, randomised controlled trials that are able to detect any treatment effect in the context of congenital heart surgery that is performed nowadays with a very low morbidity and mortality. These trials will need to be similar in design to the large steroid trials in adult cardiac surgery [28].

## Are steroids worth the risk?

Because of the lack of adequately powered studies, it is difficult to assess the short-term risk of steroid administration. However, several large registry retrospective studies of children undergoing heart surgery found no effect of steroids on clinical outcomes; furthermore, some raised concerns about infection in the steroid-treated groups [29–32]. Notably, the Pasquali et al. study retrospectively analysed a population of almost 46,730 children undergoing heart surgery and found no significant benefit of steroids. Furthermore, the steroids were associated with increased morbidity in the lower risk patient groups [29]. In other patient groups, there is emerging evidence that steroids have potential risks. A recently published retrospective study of more than half a million adults that received a short-term course of steroids found an increase in the rates of sepsis, venous thromboembolism, and fractures [33]. One RCT in children with premature lung disease found early administration of steroids to have a detrimental effect on neuromotor and cognition of outcomes if children were followed up to school age [34]. Another RCT in a similar group of patients with respiratory distress found steroids to be associated with increased incidence of cerebral palsy and developmental delay [35].Therefore, there is a need to design studies with long-term follow-up aimed at measuring effect of steroids on neurocognition.

## Steroids and the hypothalamic-pituitary-adrenal axis

In the context of congenital heart surgery, steroids are given not only to blunt inflammation but also as supplementation for what is called *relative adrenal insufficiency* [36]. The pathogenesis of this entity is indeed a matter of intense debate, and as a consequence, there are no agreed diagnostic criteria [36]. In the context of critical illness, most of the diagnostic tests rely on assessing the cortisol levels measured at a few time points and the response to a synthetic adrenocorticotropic hormone (ACTH) injection [37]. Nowadays, there is an increased recognition of the importance of biological clocks and hormone rhythms necessary for adequate physiological regulation [38]. Underlying the well-known circadian cortisol rhythm there is a fine, ultradian cortisol pulsatility [39]. Therefore, such frequent variations in the cortisol concentration make the ACTH tests inaccurate because they rely on a just few time-point measurements that can be taken either at the peak or trough of the pulse [40]. Several studies tried to assess the HPA axis during surgery [41–51]. Some used a few time cortisol measurements or just ACTH stimulation tests and tried to correlate their findings with clinical outcomes. In addition to limitations of the ACTH tests already described, some of these studies use variable ACTH test doses making interpretation more difficult [52]. Moreover, a major, common limitation of most of the studies that tried to characterise relative adrenal failure in children undergoing heart surgery is that these patients received steroids at induction making impossible an accurate assessment of the HPA axis.

Certainly, there is a need for studies aimed at understanding the basic HPA axis physiology during surgery. Such studies will need to be able to characterise cortisol pulsatility by using frequent cortisol measurements during the perioperative period, in children of various ages, that have not received any perioperative steroids. Once we understand HPA axis function during surgery, more studies aimed at the correlation of the hormone rhythms with clinical endpoints are required.

## Steroids, neuroprotection, and impact on neurocognitive outcomes

Steroids are still used to treat brain oedema associated with brain tumours and were used for a long time in the treatment of head injuries until the large CRASH trial demonstrated that they can have detrimental effects [53–55]. Naturally, steroids are being used in cases with deep hypothermic circulatory arrest (DHCA) to provide neuroprotection [5]. However, the evidence in this area is only limited to in vitro [56] or in vivo, animal studies [57, 58]. Two studies used piglet models of DHCA arrest to assess the effect of steroids on brain injury. While one study found benefit [58], the other one found steroid administration to be detrimental [57]. These studies used different steroid regimens, DHCA experimental protocols, and methodologies to assess neuroprotection thus making difficult to draw any conclusions. The limited evidence in this high-risk group warrants the need for more clinical research powered to measure the effect of steroids on neuroprotection.

## Conclusions

In the current review, we discussed the evidence for steroid use in paediatric cardiac surgery in the three main indication areas: effect on inflammation and clinical outcomes, the effect on adrenal function, and on neuroprotection. Due to the difficulty in characterising the complex inflammatory response to surgery, the current studies aimed to correlate the inflammatory effect of steroids with clinical outcomes remain limited. There is a lack of large randomised controlled trial powered to look at clinical outcomes. While there are some large registry studies looking at the effect of steroids on clinical outcomes, these remain limited by their retrospective design. Despite a wealth of data on the use of steroids in paediatric heart surgery, the current studies are conflicting and have many limitations thus leaving clinicians without any guidance. There is a limited understanding of the HPA axis physiology during the perioperative periods; therefore, we do not have an adequate diagnostic test to diagnose relative adrenal failure. We do not know if this entity even exists. Most of the studies aimed at assessing HPA axis during cardiac surgery are undertaken in patients already receiving steroids and use few cortisol measurements over long periods of times. Due to such limitations, we are unable to characterise the dynamic nature of the HPA axis response during the perioperative periods and to understand if steroids do play a role or not. The future research studies should aim at measuring cortisol levels at very frequent intervals to define the HPA axis function in the perioperative period. The least studied effect of steroids is on neuroprotection during DHCA surgery, and more research aimed at this high-risk group is needed.
